# *EUF1* – a newly identified gene involved in erythritol utilization in Yarrowia lipolytica

**DOI:** 10.1038/s41598-017-12715-7

**Published:** 2017-10-02

**Authors:** Dorota A. Rzechonek, Cécile Neuvéglise, Hugo Devillers, Waldemar Rymowicz, Aleksandra M. Mirończuk

**Affiliations:** 1 Department of Biotechnology and Food Microbiology, Wroclaw University of Environmental and Life Sciences, Chelmonskiego 37, Wrocław, 57-630 Poland; 2 Micalis Institute, INRA, AgroParisTech, Université Paris-Saclay, 78350 Jouy-en-Josas, Paris, France

## Abstract

The gene *YALI0F01562g* was identified as an important factor involved in erythritol catabolism of the unconventional yeast *Yarrowia lipolytica*. Its putative role was identified for the first time by comparative analysis of four *Y. lipolytica* strains: A-101.1.31, *Wratislavia* K1, MK1 and AMM. The presence of a mutation that seriously damaged the gene corresponded to inability of the strain *Wratislavia* K1 to utilize erythritol. RT-PCR analysis of the strain MK1 demonstrated a significant increase in *YALI0F01562g* expression during growth on erythritol. Further studies involving deletion and overexpression of the selected gene showed that it is indeed essential for efficient erythritol assimilation. The deletion strain *Y. lipolytica* AMM∆euf1 was almost unable to grow on erythritol as the sole carbon source. When the strain was applied in the process of erythritol production from glycerol, the amount of erythritol remained constant after reaching the maximal concentration. Analysis of the *YALI0F01562g* gene sequence revealed the presence of domains characteristic for transcription factors. Therefore we suggest naming the studied gene *E*rythritol *U*tilization *F*actor – *EUF1*.

## Introduction

*Yarrowia lipolytica* is an unconventional yeast with high potential for use in industry. One of the most interesting metabolites is erythritol, a four-carbon polyol, produced under hyperosmotic conditions. Erythritol gained interest because of its application as a sweetener, which not only provides no calories to the human body, but may also prevent development of caries. It occurs naturally in small amounts in foods such as honey, dairy products and fruits. For the industrial scale it is produced by yeasts such as *Moniliella pollinis* and *Torula* sp., mostly from glucose^1^. *Y. lipolytica* can synthesize erythritol from glucose, though the preferred substrate is glycerol^2^. Moreover, it can utilize crude glycerol without any prior purification^3^. The conditions necessary for efficient production are low pH and high osmotic pressure of the environment, induced by high concentrations of substrates or salt^4^. An impediment to this process is the ability of *Y. lipolytica* to utilize erythritol as a carbon source. Once the initial substrate is depleted, concentrations of secondary metabolites, including erythritol, begin to fall rapidly. In the case of batch reactors, the delay in termination of culture can result in a significant decrease in productivity.

**Figure 1:**
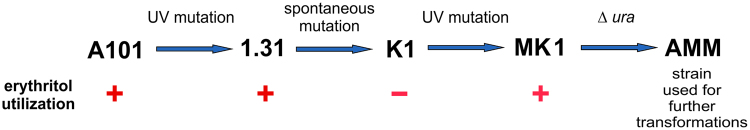
Background of *Yarrowia lipolytica* strains used in the study.

Erythritol catabolism has been subjected to the most in-depth research in the case of the bacteria *Brucella* spp., where erythritol utilization is considered responsible for triggering the virulence that leads to fetus abortion of farm animals^5^. The genes organized in the *EryABCD* operon encode erythritol kinase (EryA), which converts erythritol to L-erythritol-4-phosphate^6^, two putative dehydrogenases (EryB and EryC) and a repressor (EryD)^7^. The knowledge of erythritol utilization in yeast is much poorer, although some studies have been conducted for *Lipomyces starkeyi* ^8^. The opportunity to approach the problem from another perspective came from observations of phenotypes of a few *Yarrowia lipolytica* strains from the Department of Biotechnology and Food Microbiology at Wroclaw University of Environmental and Life Sciences (Poland). The wild strain *Y. lipolytica* A101 was isolated from soil^9^. The A-101 derivative strains 1.31, *Wratislavia* K1 and MK1 were obtained as a result of a series of UV and spontaneous mutations (Fig. 1). The most interesting phenotype was observed in *Wratislavia* K1 (hereinafter referred to as K1), which was created as a result of spontaneous mutation of strain 1.31. The mutation occurred during continuous citric acid production from glucose in a nitrogen-limited chemostat^10^. In contrast to the parental strain 1.31, which was used mainly for citric acid production, K1 was a better erythritol producer^11^. The most characteristic feature of the latter strain was its inability to utilize erythritol once produced, while grown on an erythritol synthesis medium. K1 was subjected to UV mutagenesis to further improve production parameters. The resulting strain MK1 is characterized by very low by-product formation, but it also recovered the ability to utilize erythritol^12^. The interesting properties of strains 1.31, K1 and MK1 were the rationale for full genome sequencing and mapping.

The aim of this study was to identify genes responsible for erythritol utilization in *Y. lipolytica*.

## Results

### Mutations in gene *YALI0F01562*g

**Figure 2:**
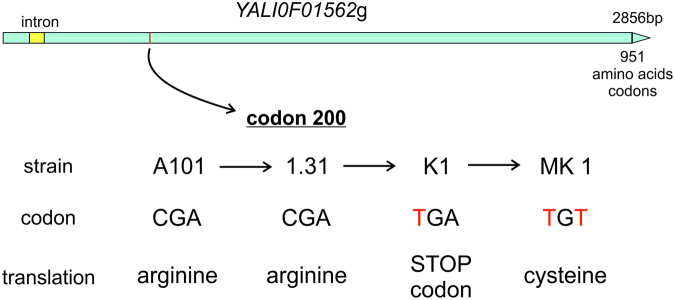
Successive mutations in gene *YALI0F01562g*.

Comparative analysis of *Y. lipolytica* strains 1.31, K1 and MK1 genomes against A101 scaffolds indicated the existence of 18 mutations between strains 1.31 and K1 and an additional 13 mutations between K1 and MK1. Particularly interesting progression of changes was noted in the gene *YALIA101S02e22540*, a homolog of *YALI0F01562g* in the genome of *Yarrowia lipolytica* strain CLIB122. Because of the greater dissemination of CLIB122 annotation, its gene name will be used in the following description of the results. *YALI0F01562*g encodes a protein of 951 amino acids. The gene carries at the 5′ end a small intron of 78 bp. A first mutation appeared in strain K1. In the first position of codon 200 there was a transition of cytosine to thymine that changed the arginine codon CGA to a terminal codon TGA (stop). In strain MK1 this mutation was also present. However, in the third position of the same codon, another point mutation occurred. An adenine to thymine transversion changed the stop codon into a cysteine codon, TGT (Fig. 2). With the restoration of the gene, strain MK1 recovered the ability to utilize erythritol. Thus, we decided to further investigate *YALI0F01562g*.

### Comparative analysis with yeast databases

Comparing *YALI0F01562g* translated into the protein sequence with the NCBI databases via the BLASTp algorithm showed that the protein contains sequences resembling two putative conserved domains: a GAL-4 like binuclear cluster DNA-binding domain at the beginning of the sequence (approximately codons 20–65) and a fungal specific transcription factor domain (codons 279–560)^13,14^. Search for a homology to whole known proteins showed some similarity to the C6 transcriptional factor from *Rhodotorula toruloides*, and to a pathway-specific nitrogen regulator from *Moniliophthora rorer*i and *Cryptococcus neoformans*. However, the highest similarity was recorded for hypothetical proteins from *Lipomyces starkeyi* NRRL Y-11557 (Table S1). Because of these results we preliminarily described the gene as *EUF1*, standing for **E**rythritol **U**tilization **F**actor, and this name was used in the nomenclature of modified strains.

### Transcript level studies

**Figure 3:**
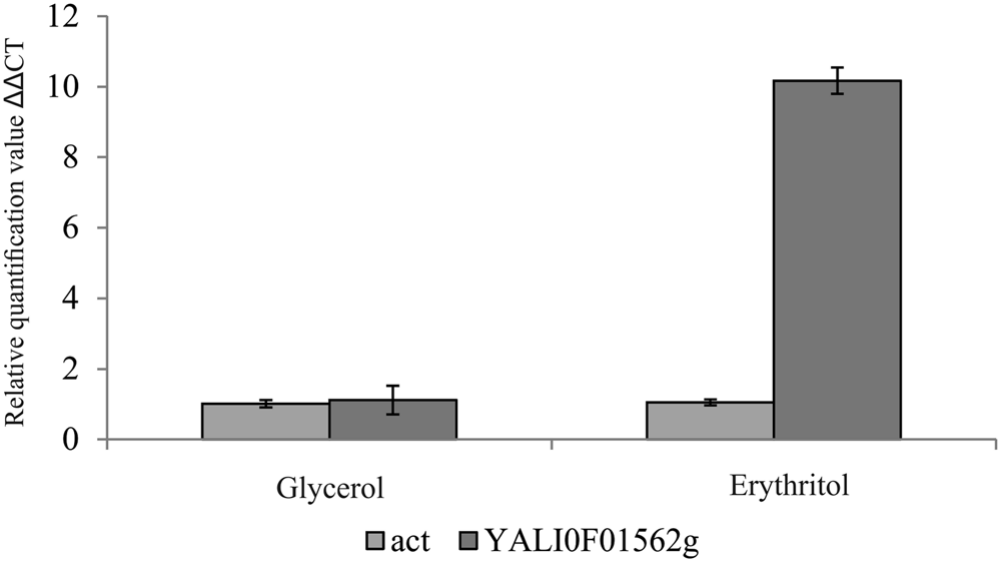
Relative expression of *YALI0F01562g* on media with glycerol or erythritol as a sole carbon source, with glucose medium used as a reference. Actin was used as a reference gene. Samples were analysed in triplicate, and the standard errors were estimated using Illumina Eco software.

The function of the gene *YALI0F01562g* was first investigated by measuring its transcript level on different carbon sources: glucose, glycerol or erythritol. Samples for RNA isolation were collected in 24-h cultures. The references for qRT-PCR were samples from glucose cultures. Growth on glycerol did not modify the expression of *YALI0F01562g*; transcripts for glycerol and glucose samples were identical (Fig. 3). In contrast, when erythritol was used as a sole carbon source, the expression of *YALI0F01562g* rose tenfold. These results showed that *YALI0F01562g* plays a role in erythritol metabolism, since its expression was significantly enhanced in the presence of this polyol. Therefore, the next step was an investigation of its influence on *Y. lipolytica* phenotype.

### Growth on different carbon sources

**Figure 4:**
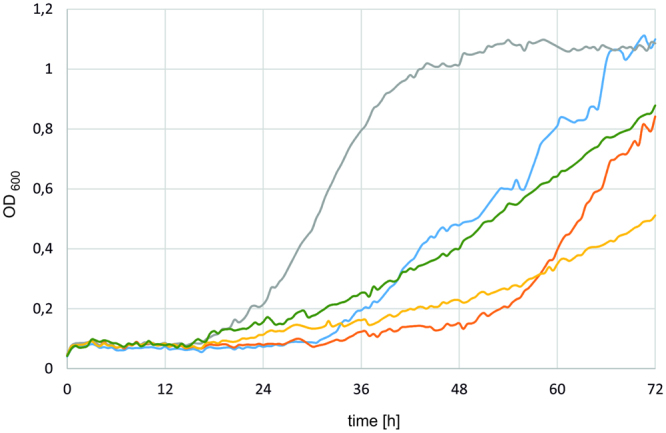
Growth curves of *Y. lipolytica* strains: MK1 (blue), K1 (orange), AMM ∆euf1 (yellow), AMM pAD-euf1 (grey) and AMM C-euf1 (green). The strains were grown on YNB supplemented with 5% erythritol as the sole carbon source. Experiment was performed at 28 °C under constant agitation using Bioscreen C.

The next stages of the work were deletion, overexpression and complementation of the *YALI0F01562g* gene. Transformants of *Y. lipolytica* for deletion (AMM ∆euf1), overexpression (AMM pAD-euf1) and complementation (AMM C-euf1) were tested for their ability to grow on different carbon sources, i.e. glucose, erythritol and glycerol using Bioscreen C. In the course of transformations the strains regain prototrophy for uracil; thus their growth can be compared. Strains MK1 and K1 were used as controls. As seen in Fig. 4, differences in growth rate were observed when erythritol was used as the sole carbon source. However, the significance of these changes was strongly influenced by the method of inoculum preparation. *Y. lipolytica* is known for lipid accumulation, so cells used as inoculum were cultivated in medium providing only a minimal amount of nutrients (YNB). If the inoculum cells were grown in rich medium such as YPD the growth rate of all strains was similar, probably due to the use of accumulated back-up materials (data not shown). Therefore, to ensure that observed growth was a result of only erythritol assimilation, the inoculum was prepared for 7 days (two cultures lasting 48 hours and one 72 hours in YNB + 2% glucose). After such treatment all strains had a long lag phase. The growth of strain AMM pAD-euf1 started at around 18 hours, and it was the only strain that reached the stationary phase by the end of the experiment. The growth of MK1 started 12 hours later, and its lag phase was longer than all modified strains, but it finally reached the highest biomass production. The slowest growth was observed for the K1 strain, which had a lag phase of more than 48 hours. However, after that time exponential growth occurred. This result was unexpected as K1 was previously believed not to utilize erythritol. The growth of the AMM ∆euf1 strain was also impaired, though the pattern was different. The growth started after 18 hours and was very slow but linear (Fig. 4).

The complementation strain AMM C-euf1 with a copy of the *YALI0F01562g* gene under the UAS1B_16_-TEF promoter displayed improved growth, but despite the shorter lag phase it reached a lower OD_600_ value than MK1. Similar experiments were performed for glucose and glycerol as the sole carbon sources, but there were no significant changes in growth of the tested strains (data not shown).

### Utilization of erythritol

**Figure 5:**
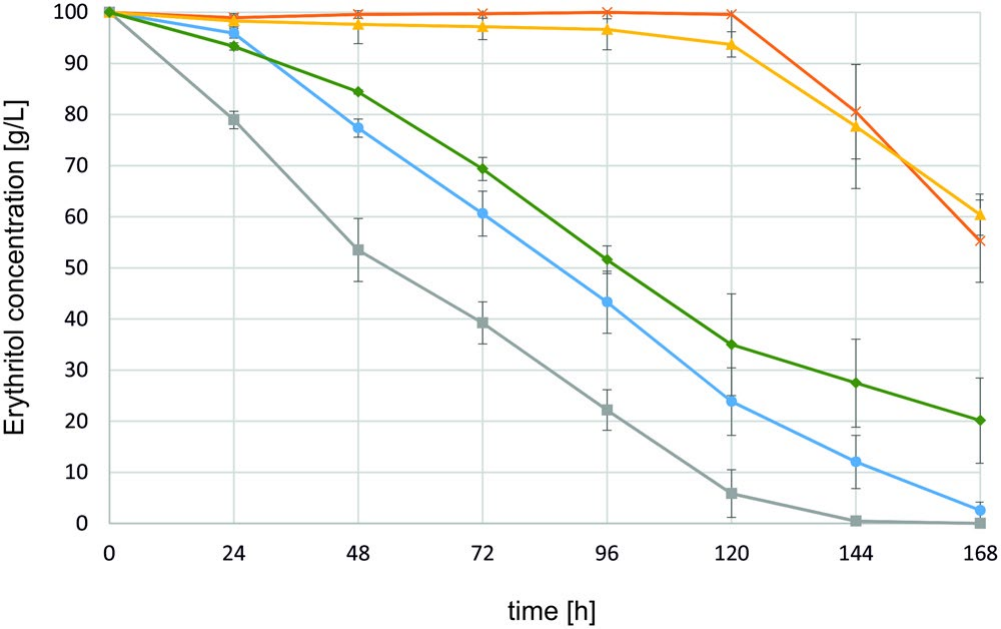
Utilisation of erythritol by *Y. lipolytica* strains MK1 (blue), K1 (orange), AMM ∆euf1 (yellow), AMM pAD-euf1 (grey) and AMM C-euf1 (green). The strains were grown in YNB medium supplemented with 10% erythritol.

The next step was to determine the erythritol utilization ratio. The initial erythritol concentration was 100 g/L in a working volume of 50 mL. During the first 24 hours of the experiment the strain AMM pAD-euf1 exhibited a significantly larger decrease in erythritol concentration (Fig. 5) than the other strains. This 24-hour advantage over the MK1 strain remained visible until complete depletion of the substrate. The complementation strain AMM c-euf1 displayed a slightly worse utilization ratio than MK1, even though it was slightly more effective during the first 24 hours.

Strains K1 and AMM ∆euf1 were also able to utilize erythritol, but the utilization of the substrate occurred only after 120 hours. Very unusual growth was observed in AMM ∆euf1 cultures; namely, the glass walls of the flasks were covered by a thick layer of biomass, while the medium beneath remained clear (Supplementary data). The efficient utilization started after the biomass layer was shaken off into liquid medium. Biofilm formation was observed also in K1 cultures, but to a smaller degree. In strains capable of fast erythritol utilization, cell growth occurred only in the liquid medium.

**Figure 6:**
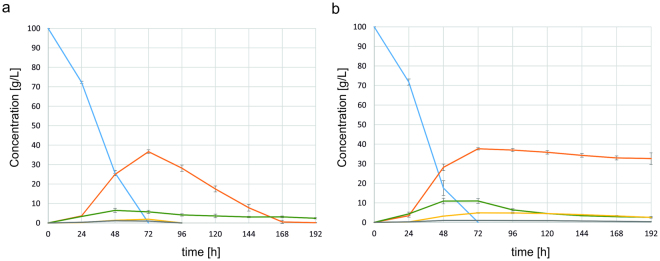
Assimilation of glycerol (blue), production of erythritol (orange), citric acid (green) and mannitol (yellow) and arabitol (grey) by strains MK1 (**A**) and AMM ∆euf1 (**B**) during the growth on erythritol production medium.

Finally, the impact of *YALI0F01562g* deletion on erythritol production was investigated during a shake-flask experiment on the Erythritol Synthesis Medium (ESM medium) (Fig. 6). The first 72 hours of culture proceeded similarly for both *Y. lipolytica* AMM ∆euf1 and MK1. By this time, glycerol, the main carbon source, was already depleted and the concentration of erythritol reached its maximum value of 37.6 ± 0.6 g/L for AMM ∆euf1 and 36.6 ± 1.0 g/L for MK1. In both cases mannitol and citric acid were formed as by-products; their concentration was higher for the AMM ∆euf1 strain. At the 96th hour, the concentration of erythritol in the MK1 culture decreased to 28.1 ± 1.6 g/L, and by the 168th hour all the polyols were utilized. In AMM ∆euf1 culture the amount of erythritol remained high, i.e. 37 ± 0.8 g/L at 96 h and 32.6 ± 3.0 g/L at 192 h. Mannitol was also still present, in the concentration of 2.5 g/L. The final amount of citric acid, 2.6 g/L, was similar to that in MK1. In both cultures a small amount of arabitol was detected (in a maximal concentration of 1.14 g/L). It was quickly utilized by MK1, but remained in AMM ∆euf1 at a final concentration of 0.37 g/L.

The maximal erythritol productivity of the MK1 strain was 0.51 g/L/h with yield 0.37 g/g after 72 hours of culture. When the experiment was prolonged to 96 hours, the yield value decreased to 0.28 g/g. The maximal productivity of the AMM pAD-euf1 strain was 0.52 g/L/h and yield 0.38 g/g after 72 hours of culture. The extension of culture to 96 h did not change the yield of erythritol.

## Discussion

Until now no genes involved in erythritol catabolism in yeasts were known. The identification of *YALI0F01562g* was possible through comparative genomic analysis and the occurrence of mutations in strains K1 and MK1. The deletion of the gene in a ura^-^ derivative of MK1 led to formation of a strain with phenotypic features more similar to K1. Strains AMM ∆euf1 and K1 share the feature of delayed induction of erythritol utilization, which significantly impaired growth on erythritol as a sole carbon source (Figs 4, 5).

The complementation strain AMM C-euf1, where *YALI0F01562g* was first deleted from its native locus and later introduced under the UAS1B_16_-TEF promoter, attested that impaired erythritol utilization was a result of *YALI0F01562g* deletion. After complementation the ability to grow and utilize erythritol was recovered, but it was slightly worsened compared to MK1 (Figs 4, 5). This may be a result of weakening of the strain by a series of transformations (*YALI0F01562g* deletion, resorting of auxotrophy and insertion of the UAS1B_16_-TEF *YALI0F01562g* cassette).

The experiments presented in this study demonstrate the importance of *YALI0F01562g* for erythritol catabolism. However, its exact role is still unclear. Analysis of the primary structure of the encoded protein indicated the presence of a DNA-binding domain and fungal specific transcription factor domain. It strongly suggests that *YALI0F01562g* may be a transcription factor rather than an enzyme directly involved in erythritol transformations, which is also indirectly implied by the obtained results.

The proteins responsible for erythritol catabolism are unlikely to be consistently present in yeast cells. This is suggested by the relatively long lag phase of cultures on erythritol utilization medium (Fig. 5). After 24 hours of MK1 growth, only a slight change in erythritol concentrations was observed. At the same time, the MK1 strain grown on glycerol or glucose as the sole carbon sources is able to utilize up to 25% of the initial substrate (data not shown). This delay may be caused by the necessity of synthesizing the required enzymes, and, if so, their formation must be induced by some intracellular signal. Thus, the disorder of the regulation system may result in the observed phenotypes of K1 and AMM ∆euf1 strains. Initially, the K1 strain was considered as unable to catabolize erythritol. However, further research demonstrated that induction of the process is possible, but with a significant delay compared to strains with a functional *YALI0F01562g* gene.

If there was an enzyme deletion, the metabolic pathway should be blocked. Some enzymes have a few orthologs that catalyze the same reaction, but if that was the reason, the existence of the orthologous gene should have been detected during BLAST analysis.

We would also like to underline the unusual growth of the AMM ∆euf1 strain in the shake flask cultures when erythritol was applied as the sole carbon source. Initially the cells were able to grow in biofilm on the vessel walls, but not in the liquid medium. Only after this step did efficient erythritol utilization occur. Biofilm growth in yeasts is associated with presence of specific transcriptional factors, such as *Bcr1* in *Candida albicans* ^15^. Their activity leads to significant morphological and physiological changes of the cells. Thus, we suspect that an unknown factor, other than *YALI0F01562g*, may influence erythritol catabolism, but its activation requires specific conditions or prolonged time.

The suggestion that *YALI0F01562g* might have broader influence than erythritol catabolism alone comes from the observation of AMM ∆euf1 growth on erythritol production medium (Fig. 6b). At the end of the experiment, aside from erythritol, there were two other polyols present in the supernatant: mannitol and arabitol. All these compounds were completely depleted by the MK1 strain. These results imply that *YALI0F01562g* should be further investigated for its role in catabolism regulation of the whole polyol group. The deletion did not have an influence on glycerol assimilation, but the catabolism of this polyol might undergo more complicated control, while it involves the enzymes of the glycolysis pathway^16^.

The deletion of *YALI0F01562g* induced the occurrence of AMM ∆euf1 phenotype resembling K1. However, on erythritol utilization medium the strains displayed different patterns of growth. The main reason is still unknown, although there are some possible explanations. First there were more mutations between K1 and MK1 strains, and their influence has not been examined yet. Secondly, the stop codon in the *YALI0F01562g* gene of K1 appeared at position 200, after the DNA binding site.

These data provide an insight into the role of *YALI0F01562g* in *Y. lipolytica* metabolism, and due to these interesting results the research is still in progress. Moreover, the obtained data are already important for industrial applications. The deletion strain AMM ∆euf1 synthesizes erythritol comparatively to MK1, but the defect in its simultaneous utilization may lead to higher erythritol concentration. Moreover, the deletion of *YALI0F01562g* may be combined with other genetic modifications, such as overexpression of genes involved in glycerol assimilation^16^, to further improve production parameters.

## Conclusion

Our study is the first work dedicated to the regulation of erythritol utilization in yeasts. A putative function of the *YALI0F01562g* gene in erythritol catabolism was proposed. However, due to the high complexity of regulation pathways, the determination of its full role requires further studies. Thus, we would like to suggest the name *EUF1* – for **e**rythritol **u**tilization **f**actor. We believe that our work will allow for a better understanding of the metabolic regulation of erythritol, not only in *Yarrowia lipolytica* but more broadly in other yeasts*. EUF1* shows some similarity to a number of proteins present in other yeasts, whose role has so far remained unknown or putative. Occurrence of the *EUF1* homolog in the only species of yeast which was previously tested for erythritol utilization indicates that its function may be universal in a large group of fungi. The study should also undoubtedly influence the optimization of erythritol production by *Yarrowia lipolytica*.

## Methods

### Microorganisms

Strains used in this study were *Y. lipolytica* A1.31, K1, MK1 and AMM, which is a *∆ura3* derivative of MK1 (Table S2). These strains belong to the Department of Biotechnology and Food Microbiology at Wroclaw University of Environmental and Life Sciences, Poland.

### Media and culture conditions

*Escherichia coli* strains were cultivated in LB medium according to standard protocols^17^. Rich yeast extract peptone glucose (YPD) medium, containing 1% (w/v) yeast extract, 1% (w/v) peptone and 2% (w/v) glucose, was used to obtain yeast biomass for DNA extraction and inoculum preparation. Medium containing YNB without amino acids (Sigma-Aldrich) supplied with 2% (w/v) glucose was used for yeast inoculum preparation.

During shake-flask experiments the cultures were grown in 0.3 L baffled flasks containing 0.03 L or 0.05 L medium on a rotary shaker (CERTOMAT IS, Sartorius Stedim Biotech) at 28 °C and 240 rpm. Erythritol utilization rate was examined on medium containing YNB and 5% (w/v) or 10% (w/v) erythritol (YNB-e medium). Erythritol synthesis was conducted in Erythritol Synthesis Medium (ESM medium) containing 100 g/L glycerol (Chempur, Poland), 2.3 g/L (NH_4_)_2_SO_4_ (Chempur), 1 g/L MgSO_4_ × 7H_2_O (Chempur), 0.23 g/L KH_2_PO_4_ (Chempur), 26.4 g/L NaCl (Chempur), 1 g/L yeast extract (Merck, Germany) and 3 g/L CaCO_3_, pH 3.0. CaCO_3_ was added separately to each flask after establishing pH 3 in order to prevent a fall of pH value.

### Bioscreen C

The yeast strains were grown in 100-well plates in 150 μL of YNB supplemented with glucose 5% (w/v), erythritol 5% (w/v) or glycerol 5% (w/v). First, the strains were grown for 24, 48 or 72 h or in YNB medium containing 2% (w/v) glucose. The inoculum was grown two or three times on YNB medium to ensure that yeast cells did not accumulate nutrients. Finally the cells were inoculated to an OD_600_ value of 0.15 in each well. Quintuple experiments were performed at 28 °C under constant agitation with a Bioscreen C system (Oy Growth Curves Ab Ltd., Finland). Growth was monitored by measuring the optical density at 420–560 nm every 30 min for 72 h.

### Sequencing

Genomic DNA was extracted with the EURX Bacterial & Yeast Genomic DNA Purification Kit (EurX, Poland) from overnight cultures of selected strains, grown on YPD medium. Sequencing was performed with the Illumina MiSeq DNA sequencing platform (paired-end [PE] 2 × 250 bp).

### Gene mapping and SNP analysis

The raw reads of each sequenced strain were trimmed with Trimmomatic version 0.32^18^ and cutadapt version 1.8.3^19^. The clean reads were mapped with BWA version 0.7.12^20^ against the *Yarrowia lipolytica* A101 reference genome^21^. Single nucleotide polymorphism (SNP) or insertions and deletions (Indels) were identified on the basis of the mpileup files generated by SAMtools version 1.2^22^. The position of the SNPs and INDELs within the chromosome was visualized with Artemis (version 16.0.0) in order to select mutations located inside a coding DNA sequence (CDS). Candidate CDSs and their protein transcripts were compared via BLAST algorithms on the National Centre of Biotechnology Information (NCBI) and Genome Resources for Yeast Chromosomes (GRYC) websites.

### RNA isolation and qRT-PCR

The shake flask cultures were grown for 24 hours in YNB medium supplemented with a 5% (w/v) carbon source, i.e. glycerol, glucose or erythritol. Next, the cultures were collected and centrifuged for 5 min at 12,000 g. The RNA was extracted using the Total RNA Mini Plus kit (A&A Biotechnology, Poland). Each sample was treated with DNase I (Thermo Scientific) according to the manufacturer’s instructions. RNA quantities were measured using a Biochrom WPA Biowave II spectrophotometer (Biochrom Ltd., UK) equipped with a TrayCell (Hellma Analytics, Germany), and the samples were stored at −80°C. Synthesis of cDNA was conducted using Maxima First Strand cDNA. Synthesis kits for RT-qPCR (Thermo Scientific) were used according to the manufacturer’s instructions. qRT-PCR analyses were carried out using a DyNAmo Flash SYBR Green qPCR Kit (Thermo Scientific) and the Eco Real-Time PCR System (Illumina, USA).

Primers for RT-PCR were designed for the gene *YALI0F01562g*, which has one intron at the beginning of the sequence. Primers qF01562-F and qF01562-R bind to the first (nt 28 in the gene sequence) and second exon (nt 283), respectively, resulting in a 178-bp qRT-PCR product. The results were normalized to the actin gene *YALI0D08272*g amplified with primers ACT-F/ACT-R and analyzed using the ddCT method^23^. Samples were analyzed in triplicate.

### Cloning and transformation protocols

All restriction enzymes were purchased from FastDigest Thermo Scientific and all of the digestions were performed according to standard protocols. The PCR reactions were set up using recommended conditions and Phusion high-fidelity DNA polymerase (Thermo Scientific). The ligation reactions were performed for 10 min at room temperature using T4 DNA Ligase (Thermo Scientific). The gel extractions were performed using the Gel Out extraction kit purchased from A&A Biotechnology (Poland). The *E. coli* minipreps were performed using the Plasmid Mini Kit (A&A Biotechnology). Transformation of *E. coli* strains was performed using standard chemical protocols^17^.

For transformation of *Yarrowia lipolytica* only strains with auxotrophy for uracil were used.

Transformation was performed according to the lithium acetate method^24^ and transformants were plated out on selective media without uracil. They were analyzed for proper integration by gDNA extraction and PCR amplification with two primer pairs. Genomic DNA (gDNA) was extracted from *Y. lipolytica* using the Genomic Mini AX Yeast Spin kit (A&A Biotechnology, Poland).

### Construction of *EUF1* deletion cassette

First, the plasmid pUC-ura for deletion was created. Primers ura-PmeI-F and ura-PmlI-R amplified the lox1-Ura-lox4 region from plasmid JM1133^25^. Next, the obtained PCR (1417 bp) product was phosphorylated and cloned into pUC18 digested with *SmaI*, resulting in the pUC_ura vector. Next, the *YALI0F01562*g promoter region and terminator were amplified by PCR using primers pF01562-HindIII-F and pF01562-SalI-R for the promoter region and tF01562-NotI-F and tF01562-PmeI-R for the terminator region. The PCR promoter fragment (1041 bp) was digested with *HindIII* and *SalI*, and cloned into corresponding sites of pUC_ura, yielding the pUC-ura-pF01562 vector. Then, the PCR terminator fragment was digested with *PmeI* and *NotI*, and cloned into corresponding sites of pUC-ura-pF01562, resulting in the pUC-ura-∆F01562 vector. The obtained vector was digested with *HindIII* and *PmeI* and transformed into *Y. lipolytica* AMM^26^, obtaining the *Y. lipolytica* AMM *∆euf1* strain. Proper integration was verified by gDNA extraction and PCR analysis. Sequences of all primers used in the study are listed in Supplementary Table S3.

Auxotrophy of strain AMM *∆euf1* was restored via excision using the Cre-lox recombinase system following transformation with the replicative plasmid pUB4-Cre1 (JME547).

### Construction of overexpression cassette

The gene *YALI0F01562g* was amplified from *Y. lipolytica* DNA with primers F01562-AscI-F and F01562-NheI-R, resulting in a 3030 bp PCR fragment. It was digested with the enzymes *SgsI* and *NheI* and cloned into corresponding sites of plasmid pAD^27^, carrying the UAS1B_16_-TEF promoter. The obtained plasmid pAD-F01562 was digested with *MssI* to create linear expression cassettes devoid of *E. coli* DNA and surrounded by *Y. lipolytica* rDNA for targeted integrations. It was used to transform *Y. lipolytica* AMM or *Y. lipolytica* AMM ∆F01562 ura-, resulting in strains AMM pAD-euf1 and AMM C-euf1, respectively.

## Electronic supplementary material

Supplementary Data
