# Supplementary material for: EUF1 – a newly identified gene involved in erythritol utilization in Yarrowia lipolytica
Source: Sci Rep. 2017 Oct 2;7:12507. doi: 10.1038/s41598-017-12715-7 (PMC5624910; doi:10.1038/s41598-017-12715-7)
Supplement: Supplementary file 1 — Supplementary Data [file 41598_2017_12715_MOESM1_ESM.pdf]

EUF1 – a newly identified gene involved in erythritol utilization in *Yarrowia lipolytica*

Dorota A. Rzechonek[1], Cécile Neuvéglise[2], Hugo Devillers[2], Waldemar Rymowicz[1], Aleksandra M. Mirończuk[1]

[1] Department of Biotechnology and Food Microbiology, Wroclaw University of Environmental and Life Sciences, Poland

[2] Micalis Institute, INRA, AgroParisTech, Université Paris-Saclay, 78350 Jouy-en-Josas, France

Correspondence author: aleksandra.mironczuk@upwr.edu.pl

Table S1. Results of BLASTP alignment performed by program BLASTP 2.7.0+(28)

Sequences producing significant alignments:

Description	Max score	Total score	Query cover	E value	Ident	Accession
YALIA101S02e22540g1_1 [Yarrowia lipolytica]	1969	1969	100%	0.0	99%	SEI32653.1
YALI0F01562p [Yarrowia lipolytica CLIB122]	1962	1962	100%	0.0	97%	XP_504866.1
hypothetical protein LIPSTDRAFT_6992 [Lipomyces starkeyi NRRL Y-11557]	414	414	82%	8,00E-128	35%	ODQ69342.1
hypothetical protein LIPSTDRAFT_108151 [Lipomyces starkeyi NRRL Y-11557]	410	410	80%	4,00E-126	36%	ODQ69153.1
hypothetical protein AWJ20_2312 [Sugiyamaella lignohabitans]	394	394	55%	4,00E-122	42%	XP_018737184.1
C6 transcription factor [Rhodotorula toruloides NP11]	246	288	61%	9,00E-67	32%	XP_016272687.1
hypothetical protein RTG_03037 [Rhodotorula toruloides ATCC 204091]	237	237	56%	1,00E-63	31%	EGU11021.1
hypothetical protein MELLADRAFT_85393 [Melampsora larici-populina 98AG31]	241	241	72%	3,00E-63	31%	XP_007408904.1
hypothetical protein RHOSPDRAFT_9619 [Rhodotorula sp. JG-1b]	231	231	62%	7,00E-62	31%	KWU45973.1
hypothetical protein PTTG_08536 [Puccinia triticina 1-1 BBBD Race 1]	224	274	61%	5,00E-58	32%	OAV87418.1
putative pathway-specific nitrogen regulator [Metarhizium anisopliae]	226	226	79%	1,00E-57	26%	KFG79639.1
N-terminal binuclear Zn cluster-containing/DNA binding domain-containing protein [Metarhizium robertsii ARSEF 23]	222	222	79%	2,00E-56	25%	XP_007824791.1
hypothetical protein H634G_10342 [Metarhizium anisopliae BRIP 53293]	222	222	79%	2,00E-56	25%	KJK74435.1
hypothetical protein VP01_1255g5 [Puccinia sorghi]	220	220	63%	9,00E-56	31%	KNZ62560.1
pathway-specific nitrogen regulator [Nannizzia gypsea CBS 118893]	219	219	79%	2,00E-55	26%	XP_003171056.1
putative pathway-specific nitrogen regulator [Metarhizium acridum CQMa 102]	219	219	79%	2,00E-55	26%	XP_007812127.1
Transcription factor, fungi [Metarhizium brunneum ARSEF 3297]	218	218	79%	3,00E-55	25%	XP_014543145.1
Transcription factor, fungi [Metarhizium majus ARSEF 297]	218	218	79%	5,00E-55	26%	XP_014575331.1
Transcription factor, fungi [Metarhizium guizhouense ARSEF 977]	215	215	79%	4,00E-54	25%	KID83586.1
hypothetical protein PSTG_14371 [Puccinia striiformis f. sp. tritici PST-78]	213	262	61%	1,00E-53	32%	KNE92201.1
hypothetical protein PGTG_12440 [Puccinia graminis f. sp. tritici CRL 75-36-700-3]	209	209	55%	2,00E-52	32%	XP_003330903.2
Transcription factor [Metarhizium rileyi RCEF 4871]	209	209	79%	2,00E-52	25%	OAA37614.1
hypothetical protein BBAD15_g4873 [Beauveria bassiana D1-5]	209	209	76%	5,00E-52	26%	KGQ09819.1
pathway-specific nitrogen regulator [Beauveria bassiana ARSEF 2860]	208	208	76%	1,00E-51	26%	XP_008596771.1
C6 transcription factor [Trichophyton rubrum CBS 118892]	206	206	56%	3,00E-51	28%	XP_003232178.1
Fungal specific transcription factor [Cordyceps confragosa RCEF 1005]	205	205	74%	7,00E-51	25%	OAA78577.1
C6 transcription factor [Penicillium brasilianum]	204	204	74%	1,00E-50	26%	OOQ87802.1
C6 transcription factor [Pochonia chlamydosporia 170]	203	203	77%	2,00E-50	26%	XP_018146291.1

fungal-specific transcription factor domain-domain-containing protein [Clohesyomyces aquaticus]	201	201	69%	1,00E-49	26%	ORY09819.1
Fungal specific transcription factor [Cordyceps militaris CM01]	201	201	76%	2,00E-49	26%	XP_006667382.1
hypothetical protein LY89DRAFT_576660 [Phialocephala scopiformis]	201	201	72%	2,00E-49	26%	XP_018076016.1
pathway-specific nitrogen regulator [Alternaria alternata]	199	199	67%	8,00E-49	26%	OWY44396.1
hypothetical protein PMG11_03767 [Penicillium brasilianum]	199	199	74%	9,00E-49	25%	CEO59079.1
c6 transcription [Moesziomyces aphidis DSM 70725]	197	197	73%	1,00E-48	25%	ETS64428.1
hypothetical protein GLRG_10273 [Colletotrichum graminicola M1.001]	198	198	79%	1,00E-48	25%	XP_008099149.1
hypothetical protein CC77DRAFT_981779 [Alternaria alternata]	198	198	67%	1,00E-48	26%	XP_018389997.1
hypothetical protein [Tuber melanosporum Mel28]	197	197	69%	2,00E-48	27%	XP_002840853.1
hypothetical protein PANT_15c00087 [Moesziomyces antarcticus T-34]	195	195	73%	6,00E-48	25%	GAC75458.1
putative transcriptional regulatory protein-like protein [Acremonium chrysogenum ATCC 11550]	195	195	78%	1,00E-47	25%	KFH48539.1
hypothetical protein VHEMI01959 [Torrubiella hemipterigena]	194	194	79%	3,00E-47	25%	CEJ81850.1
hypothetical protein PFICI_14901 [Pestalotiopsis fici W106-1]	193	193	58%	7,00E-47	26%	XP_007841673.1
hypothetical protein SS1G_00794 [Sclerotinia sclerotiorum 1980 UF-70]	193	193	77%	8,00E-47	25%	XP_001598705.1
hypothetical protein sscle_03g024370 [Sclerotinia sclerotiorum 1980 UF-70]	193	193	77%	9,00E-47	25%	APA07667.1
hypothetical protein M438DRAFT_362995 [Aureobasidium pullulans EXF-150]	192	192	69%	1,00E-46	27%	KEQ86827.1
hypothetical protein SEPMUDRAFT_147008 [Sphaerulina musiva SO2202]	192	192	69%	1,00E-46	26%	XP_016763140.1
hypothetical protein ASPBRDRAFT_192976 [Aspergillus brasiliensis CBS 101740]	192	192	74%	2,00E-46	24%	OJJ74242.1
hypothetical protein ASPTUDRAFT_189011 [Aspergillus tubingensis CBS 134.48]	191	191	69%	2,00E-46	26%	OJI84917.1
conserved hypothetical protein [Moesziomyces antarcticus]	190	190	72%	4,00E-46	24%	XP_014654630.1
related to pathway-specific nitrogen regulator [Rhynchosporium commune]	190	190	77%	6,00E-46	23%	CZS98992.1
hypothetical protein ASPFODRAFT_43197 [Aspergillus luchuensis CBS 106.47]	189	189	69%	8,00E-46	26%	OJZ89890.1
putative pathway-specific nitrogen regulator protein [Botrytis cinerea BcDW1]	189	189	77%	2,00E-45	25%	EMR83240.1
C6 transcription factor [Aspergillus niger CBS 513.88]	188	188	74%	3,00E-45	25%	XP_001389765.1
similar to transcription factor Cys6 [Botrytis cinerea T4]	188	188	77%	3,00E-45	25%	CCD43887.1
hypothetical protein PV06_07232 [Exophiala oligosperma]	182	182	56%	2,00E-44	26%	XP_016261917.1
hypothetical protein M436DRAFT_58451 [Aureobasidium namibiae CBS 147.97]	184	184	69%	2,00E-44	27%	XP_013422582.1
Zn2/Cys6 DNA-binding protein [Glarea lozoyensis ATCC 20868]	184	184	71%	5,00E-44	24%	XP_008086261.1
hypothetical protein V500_01908 [Pseudogymnoascus sp. VKM F-4518 (FW-2643)]	184	184	73%	6,00E-44	24%	KFY97864.1
related to pathway-specific nitrogen regulator [Rhynchosporium agropyri]	183	183	77%	7,00E-44	23%	CZS95571.1

hypothetical protein GALMADRAFT_252220 [Galerina marginata CBS 339.88]	178	178	73%	1,00E-42	26%	KDR72893.1
related to pathway-specific nitrogen regulator [Fusarium proliferatum]	179	179	75%	2,00E-42	24%	CVL02810.1
related to pathway-specific nitrogen regulator [Rhynchosporium secalis]	177	177	56%	5,00E-42	25%	CZT40832.1
hypothetical protein CALVIDRAFT_552314 [Calocera viscosa TUFC12733]	177	177	52%	6,00E-42	28%	KZP01230.1
hypothetical protein CALCODRAFT_479789 [Calocera cornea HHB12733]	176	176	49%	2,00E-41	27%	KZT61509.1
hypothetical protein SI65_08362 [Aspergillus cristatus]	176	176	69%	2,00E-41	25%	ODM16362.1
hypothetical protein DACRYDRAFT_112461 [Dacryopinax primogenitus]	174	174	46%	5,00E-41	28%	EJT96846.1
c6 transcription factor [Diplodia corticola]	174	217	67%	5,00E-41	25%	XP_020127084.1
pathway-specific nitrogen regulator [Marssonina brunnea f. sp. 'multigermtubi' MB_m1]	174	174	55%	8,00E-41	26%	XP_007289054.1
hypothetical protein OIDMADRAFT_131959 [Oidiodendron maius Zn]	173	173	62%	1,00E-40	25%	KIM96394.1
hypothetical protein ASPGLDRAFT_987039 [Aspergillus glaucus CBS 516.65]	172	172	66%	2,00E-40	25%	OJJ87721.1
hypothetical protein CYLTODRAFT_425553 [Cylindrobasidium torrendii FP15055 ss-10]	170	170	73%	3,00E-40	24%	KIY64083.1
pathway-specific nitrogen regulator [Cryptococcus neoformans var. grubii Th84]	170	170	79%	7,00E-40	25%	OXG54436.1
conserved hypothetical protein [Cryptococcus neoformans var. neoformans JEC21]	169	169	73%	8,00E-40	24%	AAW43794.2
pathway-specific nitrogen regulator [Cryptococcus neoformans var. grubii AD1-83a]	170	170	79%	9,00E-40	25%	OWZ54963.1
hypothetical protein AYX15_00266 [Cryptococcus neoformans var. grubii]	170	170	79%	1,00E-39	25%	OWZ69086.1
pathway-specific nitrogen regulator [Moniliophthora roreri MCA 2997]	169	169	72%	2,00E-39	25%	XP_007845309.1
hypothetical protein AYX14_00466 [Cryptococcus neoformans var. grubii]	169	169	79%	2,00E-39	25%	OWZ73980.1
pathway-specific nitrogen regulator [Cryptococcus neoformans var. grubii Bt1]	169	169	79%	2,00E-39	25%	OWT38020.1
pathway-specific nitrogen regulator [Cryptococcus neoformans var. grubii]	169	169	79%	2,00E-39	25%	OXH41272.1
hypothetical protein PV05_03680 [Exophiala xenobiotica]	167	167	54%	2,00E-39	26%	XP_013319800.1
pathway-specific nitrogen regulator [Cryptococcus neoformans var. grubii Ze90-1]	169	169	79%	2,00E-39	25%	OXG33193.1
hypothetical protein, variant [Exophiala xenobiotica]	167	167	54%	2,00E-39	26%	XP_013319799.1
pathway-specific nitrogen regulator [Cryptococcus neoformans var. grubii H99]	169	169	79%	2,00E-39	24%	XP_012046441.1
pathway-specific nitrogen regulator [Cryptococcus gattii VGII R265]	166	166	72%	5,00E-39	26%	KGB75272.1
pathway-specific nitrogen regulator [Cryptococcus gattii VGII LA55]	166	166	72%	6,00E-39	26%	KIR30681.1
hypothetical protein EURHEDRAFT_446483 [Aspergillus ruber CBS 135680]	168	168	49%	6,00E-39	26%	EYE98974.1
hypothetical protein AYX13_02981 [Cryptococcus neoformans var. grubii]	167	167	76%	9,00E-39	25%	OXC68529.1
hypothetical protein V499_00259 [Pseudogymnoascus sp. VKM F-103]	166	166	74%	2,00E-38	24%	KFY80926.1
hypothetical protein J056_002844 [Wallemia	164	164	47%	3,00E-38	27%	XP_009270384.1

ichthyophaga EXF-994]						
pathway-specific nitrogen regulator [Moniliophthora roreri MCA 2997]	165	165	49%	4,00E-38	28%	XP_007848971.1
Nitrogen assimilation transcription factor nirA OS=Emericella nidulans (strain FGSC A4 / ATCC 38163 / CBS 112,46 / NRRL 194 / M139) GN=nirA PE=4 SV=1 [Rhizoctonia solani AG-1 IB]	164	164	46%	7,00E-38	28%	CEL59441.1
hypothetical protein SCHCODRAFT_269952 [Schizophyllum commune H4-8]	163	163	76%	8,00E-38	25%	XP_003031293.1
pathway-specific nitrogen regulator [Kwoniella dejecticola CBS 10117]	162	209	56%	2,00E-37	25%	XP_018259761.1
hypothetical protein I302_04720 [Kwoniella bestiolae CBS 10118]	162	208	66%	2,00E-37	28%	XP_019045980.1
Transcription factor PDR1 [Rhizoctonia solani]	162	162	46%	2,00E-37	28%	CUA70286.1
pathway-specific nitrogen regulator [Kwoniella mangroviensis CBS 8507]	162	216	57%	3,00E-37	26%	XP_019000338.1
C6 transcription factor [Rhizoctonia solani AG-3 Rhs1AP]	162	162	46%	4,00E-37	28%	EUC67392.1
pathway-specific nitrogen regulator [Kwoniella mangroviensis CBS 10435]	161	217	57%	5,00E-37	26%	OCF54682.1
pathway-specific nitrogen regulator [Kwoniella mangroviensis CBS 8886]	160	216	57%	9,00E-37	26%	OCF78690.1
fungal-specific transcription factor domain-domain-containing protein [Naematelia encephala]	160	207	58%	1,00E-36	25%	ORY26020.1
hypothetical protein VE01_00388 [Pseudogymnoascus verrucosus]	160	160	72%	2,00E-36	24%	XP_018135213.1

Table S2. Strains and plasmids used in this study.

Strain	Genotype or plasmid	Comments	Source
E. coli			
DH5α	F^- endA1 glnV44 thi-1 recA1 relA1 gyrA96 deoR nupG Φ80dlacZΔM15 Δ(lacZYA-argF)U169, hsdR17(rK- mK+), λ–		(29)
DH5α	pAD, $UAS1_{B16}$TEF promoter		(27)
DH5α	pUC-ura		This study
DH5α	pUC-ura-pF01562		This study
DH5α	pUC-ura-ΔF01562		This study
DH5α	pAD-F01562		This study
Y. lipolytica			
A101	*MATA*	Wild type	(9)
1.31	*MATA*	A101 UV mutant	(10)
Wratislavia K1	*MATA*	1.31 spontaneous mutant	(10)
MK1	*MATA*	K1 UV mutant	(12)
AMM	*MATA*, ura3-302	MK1 derived	(26)
AMM pAD-*EUF1*	*MATA*, ura3-302, pAD-*EUF1*::*URA3*	AMM transformant, Euf1 overexpression	This study
AMM Δ*euf1*	*MATA*, ura3-302, euf1::*URA3*	AMM transformant, *EUF1* deletion	This study
AMM Δeuf1 ura^-	*MATA*, ura3-302, euf1	AMM Δ*euf1* transformant, *URA3* excision	This study
AMM C-euf1	*MATA*, ura3-302, euf1, pAD-*EUF1*::*URA3*	AMM Δeuf1 ura^- complemented for *EUF1* and *URA3*	This study

Table S3. Primers used in this study

**Primer**	**Sequence (5'→ 3')**
Ura-PmeI-F	GCTGTTTAAACCCACCGCGGTGGCGGCCGCTCTAG
Ura-PmlI-R	TTACACGTGGCTGGGTACCGGGCCCCCCCTC
pF01562-HindIII-F	GCGAAGCTTAGTGCTGCCGGAAACC
pF01562-SalI-R	CGCGTCGACCTACCGGCATGTTGTG
tF01562-NotI-F	TAAGCGGCCGCCGAGTAACGACATAACG
tF01562-PmeI-R	GCTGTTTAAACTCTACGGTGGTTCTG
F01562-AscI-F	ATCGGCGCGCCCCGGTAGCGATATGAACG
F01562-NheI-R	CTCGCTAGCCAATCGCGTTGCTACTATAC
qF01562-F	ACCGGAGACGTCCAGAGTTC
qF01562-R	TATCGGTGCCCTTCTTGGTG

28 Stephen F. Altschul, Thomas L. Madden, Alejandro A. Schäffer, Jinghui Zhang, Zheng Zhang, Webb Miller, and David J. Lipman (1997), "Gapped BLAST and PSI-BLAST: a new generation of protein database search programs", Nucleic Acids Res. 25:3389-3402.

29 Hanahan, D. DNA cloning: a practical approach. (IRL Press, 1985)
